# Evaluation of the “Kosish Cocktail” in Treating Severe Pain in “Home Care” in Morphine-naïve Communities

**DOI:** 10.4103/0973-1075.63130

**Published:** 2010

**Authors:** AK Dam, Nivedita Datta, Usha Rani Mohanty

**Affiliations:** Qr.2120, Sector 4 C, Bokaro Steel City, Jharkhand, India

**Keywords:** Ketamine, Kosish cocktail, Pain, Pentazocine

## Abstract

**Background:**

Inavailability of morphine continues to plague most parts of India. Good palliative care must, however, focus on resources that are locally available, culturally acceptable, financially affordable, and easily applicable. These factors were all integral to the development of the “Kosish cocktail” for use in severe pain. This cocktail is a mixture of ketamine, midazolam, pentazocine lactate, and other adjuvants for use in the domiciliary set-up as intermittent subcutaneous injections in a morphine-naïve community. Our aims and objectives were: (1) To assess the efficacy of the “Kosish cocktail” in treating severe pain in terminally ill patients; (2) To assess the safety profile and note any adverse effects; (3) To evaluate its use in domiciliary set-ups in terms of safety and efficacy; (4) To empower the patient and the family in the process of patient care.

**Materials and Methods:**

Eight patients with advanced cancer and severe pain, who were already on WHO Step II drug therapy, were enrolled for this study. The cocktail was administered subcutaneously in every four hours and SOS. Subjective and objective parameters were recorded and the data analyzed using Student’s t-test with a *P*<0.05 being considered significant.

**Results:**

There was a statistically significant improvement in the subjective parameters 12 hours after the initiation of therapy, except for the persistence of fatigue.

**Conclusions:**

On the basis of this qualitative study, the authors confirm the efficacy and safety of the use of the Kosish cocktail in treating severe pain, and strongly recommend it for newly started hospices, especially for use in the domiciliary set-up.

## INTRODUCTION

Inavailability of morphine remains a nagging problem in most parts of India. Good palliative care should be easily available, easily accessible, culturally acceptable, and at a cost which the community can easily afford. These basic principles have been adopted in designing the “Kosish cocktail” for patients of the WHO step III of the analgesic ladder in a morphine-naive community.

Pentazocine lactate is an agonist-antagonist opioid with a very rapid onset of analgesia, a duration of action comparable to that of parenterally administered morphine, a ceiling effect on its respiratory depressant effects, and a propensity to be hemodynamically stable. It acts by interacting with opioid receptor sites primarily in the limbic system, thalamus, and spinal cord by blocking the transmission of pain impulses. It is also readily available in most parts of India as it does not require a “special” license for stocking.

Ketamine hydrochloride is an NMDA receptor antagonist with excellent analgesic properties even at subanesthetic doses. The analgesic action of low, subanesthetic doses of ketamine predominantly derives from its activity-dependent, noncompetitive blockade of the glutaminergic NMDA receptor channel complex by binding at phencyclidine (PCP) binding sites in ion channels[1]. Also, low-dose ketamine has been found to be opioid-sparing, reduces nausea and vomiting, and has minimal side-effects as per Level I evidence.[2]

Keeping the above facts in mind, the following study was designed to assess the efficacy of subcutaneously (S/C) administered “Kosish cocktail” (a mixture of ketamine 10 mg/mL), Pentazocine lactate (10 mg/mL), midazolam (0.5 mg/mL), and Glycopyrollatr (0.4 mg), lignocaine (2 ml of 2% preservative free) as the primary drugs).

### Aims and objectives:

- To assess the efficacy of the “Kosish cocktail” administered S/C for severe pain (WHO step III) in the terminally ill
- To assess the safety profile of the cocktail
- To note any adverse effects, if any, of the cocktail
- To evaluate its use in domiciliary set-up in terms of safety and efficacy
- To empower the patient and family in the process of patient care

Study design: Nonrandomized, qualitative, prospective study initiated in a controlled environment of a critical care unit (CCU) and continued further in the domiciliary set-up.

## MATERIALS AND METHODS

A total of eight patients with advanced cancer and severe pain (VAS>7 on initial presentation) and already on WHO Step II drug therapy were enrolled in this study. Written and informed consent was obtained from all the patients. A 24 G butterfly needle was sited S/C in a suitable site on the patient. The Memorial Symptom Assessment Scale (MSAS) was used to score the symptoms on a numerical scale of 1 to 10, as described by the patients before the start of therapy, 12 hours after starting therapy and subsequently at 24, 48, and 72 hours. All the patients were admitted to a CCU for the first 24 hours after the initiation of the therapy and care was then continued in the domiciliary set-up. One milliliter of the “cocktail” was injected S/C at four hourly intervals with an additional bolus of 1 mL being allowed on an SOS basis.

In addition to the MSAS (which assessed the ‘subjective component’), the pulse, blood pressure (NIBP), oxygen saturation (SpO_2_), and respiratory rate (RR) were also noted (which assessed the ‘objective component’) at the aforementioned points of time.

## RESULTS

The following table depicts the subjective and objective parameters measured and compared with the baseline [Table 1].

**Table 1:** The subjective and objective parameters measured and compared with the baseline

N = 8	Baseline	After 12 hrs	After 24 hrs	After 48 hrs
Pain	8.63(1.3)	2.25(1.0) *P*=0.0	2.75(0.7) *P*=0.0	3.13(0.6) *P*=0.0
Appetite	8.0(0.7)	5.88(0.6) *P*=0.0	6.88(1.2) *P*=.05	7.38(1.1) *P*=.23
Sleep	7.50(1.6)	2.0(0.5) *P*=.00	3.38(0.9) *P*=.00	3.25(1.0) *P*=.00
Well being	8.63(0.9)	2.38(0.5) *P*=0.0	3.50(1.4) *P*=0.0	2.75(0.8) *P*=0.0
Fatigue	8(0.93)	6.63(1.6) *P*=.06	7.13(1.3) *P*=.18	6.63(1.5) *P*=.05
Nausea	7.88(1.2)	1.75(1.3) *P*=0.0	3.63(1.0) *P*=0.0	2.75(1.4) *P*=0.0
Depression	8.88(0.9)	2.88(1.2) *P*=0.0	3.75(1.2) *P*=0.0	3.75(1.9) *P*=0.0
Dyspnea	5.63(2.0)	1.63(0.9) *P*=0.0	3.50(0.9) *P*=0.02	3.0(1.5) *P*=0.01
SpO_2_	92.2(2.3)	92.5(1.6) *P*=0.8	93.7(2.0) *P*=0.2	92.3(1.3) *P*=0.9
Pulse	92.2(11.9)	77.0(7.1) *P*=0.01	82.8(7.2) *P*=0.08	87.9(9.8) *P*=0.06
Respiratory rate	23.3(6.2)	18.5(2.5) *P*=0.06	19.3(3.2) *P*=0.13	20.75(6.2) *P*=0.28

All the patients had a statistically significant (*P*< 0.05) improvement in their subjective parameters 12 hours after the initiation of therapy, except for the persistence of fatigue, which persisted after 24 and 48 hours after starting therapy.

The primary care-givers were satisfied with the therapy, especially with the relief it afforded to their loved ones. Also, they were happy as they could participate in the care-giving process. The patients had significant relief from pain particularly after the start of therapy, which persisted throughout the duration of the study.

The main adverse effects noted in our study, besides the problems arising from subcutaneous administration of medications, were as follows:

- Pain on injection (*n*=8): rectified by the addition of 2 mL of 2% preservative-free lignocaine in the mixture
- Nausea (*n*=7): rectified by addition of the ondansetron to the mixture; a few patients required further addition of dexamethasone.
- Dysphoria and confusion (*n*=2): required the addition of haloperidol. However, it could not be ascertained if the dysphoria resulted from drug therapy or as a part of the terminal delirium arising from other causes. Both the subjects expired 4–5 days after starting therapy, due to advanced malignancy.
- Increase in the dose of medication required over four days (*n*=4): this probably resulted from the titration of doses resulting from interindividual variability. The authors suspect the development of tachyphylaxis to ketamine to be another probable cause.

## DISCUSSION

Inavailability of morphine should not discourage providers from providing palliative care. Making use of locally available resources can go a long way in providing good symptom control. Morphine, no doubt, remains the “gold standard” but that does not necessarily mean that the agonist-antagonist group of narcotics does not have any role to play, especially in morphine-naïve communities.

As seen in this study, the use of the Kosish cocktail provided excellent symptom relief by using locally available resources at an affordable cost. Keeping in view the high incidence of nausea and vomiting in our group of patients prior to the start of therapy and the potential of ketamine and pentazocine to cause nausea, it was decided to add ondansetron routinely to the Kosish cocktail. Metoclopromide would have been a cheaper alternative but it was not miscible in the same syringe (precipitation was observed). However, a study comparing the continuous subcutaneous infusion of low-dose ketamine for postoperative analgesia with intermittent subcutaneous injections of morphine, found better analgesia with improved respiratory function as well as less sedation, nausea, and vomiting.[2]

Ketamine was found to be safe and effective in an audit of analgesia for burn dressings for adults using a PCA combining ketamine 10 mg and midazolam 0.5 mg per bolus.[2] A combination using ketamine and midazolam was found to be more safe and effective than fentanyl/midazolam or porpofol/midazolam for sedation during fracture reduction in children.[3]

The prepared mixture was found to be very stable at room temperature and had good analgesic effects even 48 hours after preparation. None of our patients had any respiratory depression as pentazocine has a ceiling effect on its respiratory depression, thus making it very safe for domiciliary use. Also, both ketamine and pentazocine are hemodynamically stable drugs, a factor which is of concern when dealing with the terminally ill. Working in a virgin community is demanding as you cannot afford to have “accidents” after initiating drug therapy for symptom control.

Ketamine preserves sympathetic reflexes that help support blood pressure in patients who have lost blood and it also does not interfere with the respiratory drive.[2]

Our method of intermittent injections delivered via a subcutaneous port was culturally acceptable because the majority of our rural and illiterate population misbelieves that “good medications” should be given as injections. This method was also acceptable to the illiterate care-givers as they were taught to inject a predetermined amount (as per the markings on the syringe) of drug. The patients were also happy by the fact that they were getting symptom control at home by the participation of the family members. This promoted “bonding”, a factor that helped both the patient and the family members psychologically and spiritually. It also promoted social bonding as curious friends and neighbors would drop by, prompted by the appearance of the palliative care team!

## CONCLUSION

On the basis of this qualitative study, the authors confirm the efficacy and safety of the use of the Kosish cocktail in treating severe pain in morphine-naïve communities. This cocktail is especially strongly recommended for newly started hospices, especially for use in the domiciliary set-up.
